# Supplementary material for: Mechanisms governing the pioneering and redistribution capabilities of the non-classical pioneer PU.1
Source: Nat Commun. 2020 Jan 21;11:402. doi: 10.1038/s41467-019-13960-2 (PMC6972792; doi:10.1038/s41467-019-13960-2)
Supplement: Supplementary file 7 — Source data [file 41467_2019_13960_MOESM7_ESM.zip › Source_Data/FigureS2/FigureS2B_MotifScanOutput/KmeansCluster3/homerResults/motif21.similar.html]

motif21

## Information for motif21

A
T
G
C
C
G
T
A
C
T
A
G
G
C
A
T
A
C
G
T
A
G
T
C
A
C
G
T
A
G
T
C
T
C
G
A
A
G
C
T
A
C
T
G
C
G
T
A
C
A
T
G
C
G
T
A
  
Reverse Opposite:  

A
C
G
T
G
T
A
C
A
C
G
T
A
G
T
C
T
C
G
A
A
G
C
T
A
C
T
G
C
G
T
A
A
C
T
G
C
G
T
A
C
G
T
A
A
G
T
C
C
G
A
T
T
A
C
G
  

|  |  |
| --- | --- |
| p-value: | 1e-47 |
| log p-value: | -1.086e+02 |
| Information Content per bp: | 1.826 |
| Number of Target Sequences with motif | 151.0 |
| Percentage of Target Sequences with motif | 3.17% |
| Number of Background Sequences with motif | 258.2 |
| Percentage of Background Sequences with motif | 0.61% |
| Average Position of motif in Targets | 102.2 +/- 43.6bp |
| Average Position of motif in Background | 101.7 +/- 73.6bp |
| Strand Bias (log2 ratio + to - strand density) | -0.1 |
| Multiplicity (# of sites on avg that occur together) | 1.32 |
| Motif File: | file (matrix) reverse opposite |

### Similar de novo motifs found

|  |  |  |  |  |  |  |  |
| --- | --- | --- | --- | --- | --- | --- | --- |
| Rank | Match Score | Redundant Motif | P-value | log P-value | % of Targets | % of Background | Motif file |
| 1 | 0.977 | A G C T T G A C C G A T A G T C T C G A A G C T T A C G C G T A C T A G G C T A G C T A A G T C G C A T | 1e-47 | -108.520379 | 2.87% | 0.49% | motif file (matrix) |
| 2 | 0.929 | C G A T T G A C G A C T A G T C C T G A A G C T T C A G C G T A A T C G C G T A C T G A A G T C | 1e-41 | -96.250714 | 3.40% | 0.80% | motif file (matrix) |
| 3 | 0.889 | T G A C G A C T A G T C C T G A G A C T T A C G C G T A A C T G G C T A C G T A A G T C | 1e-32 | -75.013193 | 3.31% | 0.96% | motif file (matrix) |
| 4 | 0.881 | A C G T A G T C T C G A G A C T A C T G G T C A A C T G C T G A C G T A A G T C | 1e-32 | -73.975654 | 2.56% | 0.59% | motif file (matrix) |
| 5 | 0.807 | A C G T G T A C A C G T A G T C C T G A A G C T A C T G C G T A | 1e-17 | -41.336461 | 2.85% | 1.13% | motif file (matrix) |
